# The potential role of PIWI-interacting RNAs in non-small cell lung cancer

**DOI:** 10.3389/fonc.2025.1660236

**Published:** 2025-09-25

**Authors:** Linqian Song, Jian-Guo Zhou, Hu Ma

**Affiliations:** ^1^ Department of Oncology, The Second Affiliated Hospital of Zunyi Medical University, Zunyi, Guizhou, China; ^2^ Key Laboratory for Cancer Prevention and Treatment of Guizhou Province, Zunyi, Guizhou, China

**Keywords:** PIWI-interacting RNAs, PIWI, NSCLC, cancer progression, biomarkers, therapeutic targets

## Abstract

Non-small cell lung cancer (NSCLC) accounts for approximately 85% of lung cancer and remains the leading cause of cancer mortality globally. The lack of early diagnostic tools and effective therapeutic targets contributes to poor outcomes for NSCLC patients. PIWI-interacting RNA (piRNA), a short-stranded non-coding RNA, interacts with PIWI proteins to epigenetically regulate gene expression. Recently, much evidence suggests that piRNAs and PIWI proteins are closely associated with NSCLC progression, including cancer cell proliferation, metastasis, chemoresistance, and stemness. Therefore, piRNAs and PIWI represent promising biomarkers and therapeutic targets, showing excellent sensitivity and specificity. However, their precise functions and potential mechanisms remain incompletely understood. Thus, it is necessary to further elucidate the mechanism of piRNAs/PIWI role and enhance clinical translation. This review synthesizes the biosynthesis and function of piRNAs, as well as the regulatory mechanisms of piRNAs/PIWI in NSCLC. Additionally, we evaluate the clinical potential of the piRNAs and PIWI as novel biomarkers and therapeutic targets in NSCLC. This provides new insights into the diagnosis and treatment of NSCLC.

## 1 Introduction

Cancer remains a major public health dilemma globally, and lung cancer is the primary cause of cancer-related deaths worldwide (1). NSCLC is the predominant form of lung cancer, accounting for 85% of all cases, whereas small cell lung cancer (SCLC) is less prevalent, accounting for 15% of all cases (2). Since the initial symptoms of NSCLC are often subtle, most patients are diagnosed at advanced stages, resulting in poor prognosis and a high mortality rate (3, 4). Despite the development of tools for early detection of NSCLC and a variety of treatments such as surgery and chemotherapy and radiotherapy, the survival rate of patients remains below 35% (5, 6). This has resulted in a failure to significantly improve patient survival and prognosis. Consequently, there is an urgent need to deepen the comprehension of NSCLC pathogenesis and tumor progression, identify potential biomarkers further, and explore new therapeutic targets.

Non-coding RNAs (ncRNAs), such as microRNAs (miRNAs), long non-coding RNAs (lncRNAs) and circular RNAs (circRNAs) play crucial roles in regulating gene expression (7). Specifically, miRNAs mainly regulate gene expression post-transcriptionally by causing mRNA degradation or translational repression (8). On the other hand, lncRNAs and circRNAs often act as competitive endogenous RNAs (ceRNAs) or provide structural support for protein complexes (9). The regulatory functions of these non-coding RNAs have been widely recognized in NSCLC. In contrast, research on piRNAs in NSCLC remains limited. However, their unique biogenesis pathway and PIWI-dependent mechanism of action confer advantages in terms of molecular stability and potential targeting specificity. Consequently, piRNAs may offer new avenues for research and clinical value in the diagnosis and treatment of NSCLC.

PIWI-interacting RNAs (piRNAs) are a class of small non-coding RNAs containing 24–31 nucleotides (nt) that have been discovered in recent years. It initially in Drosophila germ cells where they silence transposable elements to maintain genomic stability (10–12). However, subsequent research has revealed that piRNAs are also present in somatic cells and play critical regulatory roles. These include transcriptional gene silencing, modulation of mRNA stability and translation, maintenance of stem cell function, and interaction with a broad range of proteins (13, 14). Unlike other ncRNAs, the biosynthesis process of piRNAs does not depend on RNase III (Dicer) modification, and it obtains mature piRNAs mainly through two unique mechanisms: primary amplification and the ‘ping-pong’ cycle (15, 16). Notably, piRNAs often need bind to PIWI proteins (a subfamily of Argonaute proteins) to exert these unique regulatory effects and to be biosynthesized. Moreover, PIWI proteins are highly conserved across species and enhance the regulatory impact by forming the RNA-induced silencing complex (RISC) (17). The significant regulatory roles of piRNAs/PIWI have captured the interest of researchers. Recent studies have demonstrated that abnormal piRNAs and PIWI expression in NSCLC is commonly associated with tumor development (18–20). Mechanistically, abnormal piRNA expression perturbs cellular gene regulation, leading to transposable element reactivation, epigenetic reprogramming, and dysregulated expression of oncogenes or tumor suppressors, which collectively contribute to malignant transformation (21). Moreover, piRNAs and PIWI proteins regulate NSCLC occurrence and development mainly through mediating transcriptional gene silencing, post-transcriptional gene silencing, and multi-protein interactions (17, 22). These findings suggest that piRNAs and PIWI may hold potential not only as diagnostic and prognostic biomarkers but also as clinically relevant modulators. Their dysregulation correlates with therapeutic resistance, while their stability in bodily fluids highlights their promise as non-invasive indicators for liquid biopsy. Furthermore, PIWI and piRNAs themselves present novel targets for potential therapeutic interventions. However, their exact mechanisms and biological functions in NSCLC remain uninvestigated. Therefore, an intensive study of the molecular mechanisms of piRNA and PIWI in NSCLC is of great significance for their application in diagnosis, prognosis and targeted therapy. This review summarizes the biosynthesis and biological functions of piRNAs, the roles of piRNAs and PIWI proteins in NSCLC, and advances in the study of piRNAs and PIWI proteins as potential biomarkers and targets for NSCLC.

## 2 Biosynthesis of piRNA

### 2.1 Transcription of piRNA clusters

**Figure 1:**
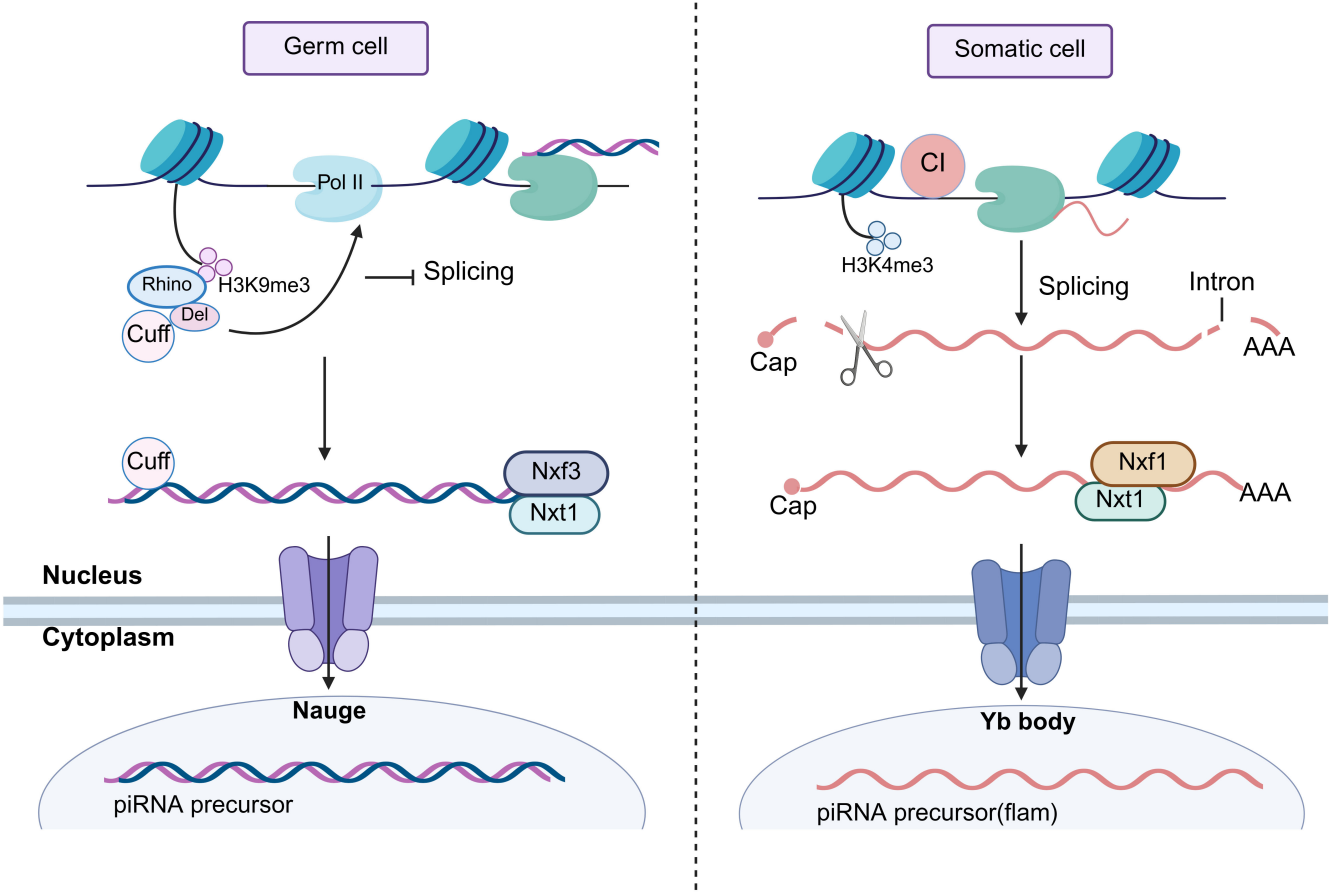
Transcription of piRNA clusters. Germ cells: double-stranded piRNA clusters lacking a promoter and containing the H3K9me3 marker. Rhino recognises H3K9me3 and recruits Deadlock and Cutoff to form the RDC complex, which activates Pol II transcription by a non-canonical mechanism. At the same time, the RDC complex prevents splicing and promotes transcriptional elongation. Subsequently, the piRNA precursor binds to the Nxf3-Nxt1 complex and is transported to nuage for processing. Somatic cells: single-stranded piRNA clusters (Flam motifs) with promoters enriched for H3K4me3 markers, dependent on CI and Pol II for canonical transcription and splicing, the products bind to Nxf1-Nxt1, and transported to Yb body via the nuclear pore complex for further processing. H3K9me3, trimethylation of histone H3 lysine 9; RDC complex, Rhino–Deadlock–Cutoff complex; Pol II, RNA polymerase II; H3K4me3, trimethylation of histone H3 lysine 4; CI, Cubitus interruptus.

Approximately 90% of piRNAs originate from specific genomic regions known as “piRNA clusters”, whose precursors are transcribed by RNA polymerase II and subsequently processed into mature piRNAs (23, 24). Based on their generation characteristics, piRNA clusters are classified as either single-stranded or double-stranded (25). Studies have shown that piRNA clusters can be widely expressed in different species, but the transcriptional mechanism of these clusters has been most intensively studied in the Drosophila germline (26, 27). Thus, studies of the Drosophila model have elucidated the transcriptional regulation of piRNA clusters (**Figure 1**). Double-stranded piRNA clusters are predominantly found in germ cells, and their transcriptional regulation is dependent on the H3K9 trimethylation tag (28–30). The Drosophila HP1 homolog (Rhino) recognises this epigenetic tag and recruits Deadlock and Cutoff to form the Rhino-Deadlock-Cutoff (RDC) complex (31, 32). It activates RNA polymerase II (Pol II)-mediated transcription, while simultaneously suppressing transcript splicing to ensure stable elongation (33, 34). Subsequently, the RDC complex recruits the Nxf3-Nxt1 complex, which facilitates the transport of piRNA precursors to the nuage for further processing (35, 36).

Furthermore, single-stranded piRNA clusters are widely distributed in somatic cells and exhibit typical Pol II-dependent transcriptional characteristics. For instance, the Flamenco (Flam) locus contains a promoter enriched with H3K4me2 marks, which drive primary transcription (37, 38). The primary transcripts of single-stranded piRNA clusters are conventionally spliced, and their transcriptional regulation is dependent on the promoter signature and the activity of the key transcription factor, Cubitus interruptus (CI) (39). The precursor piRNAs then bind to nuclear export proteins (Nxf1 and Nxt1) and are transported to the Yb body for further processing under the guidance of the nuclear pore complex (40, 41). In mammals, piRNA clusters are similarly transcribed, but their expression in germ cells and somatic cells is tightly regulated.

### 2.2 Two pathways of piRNA production

**Figure 2:**
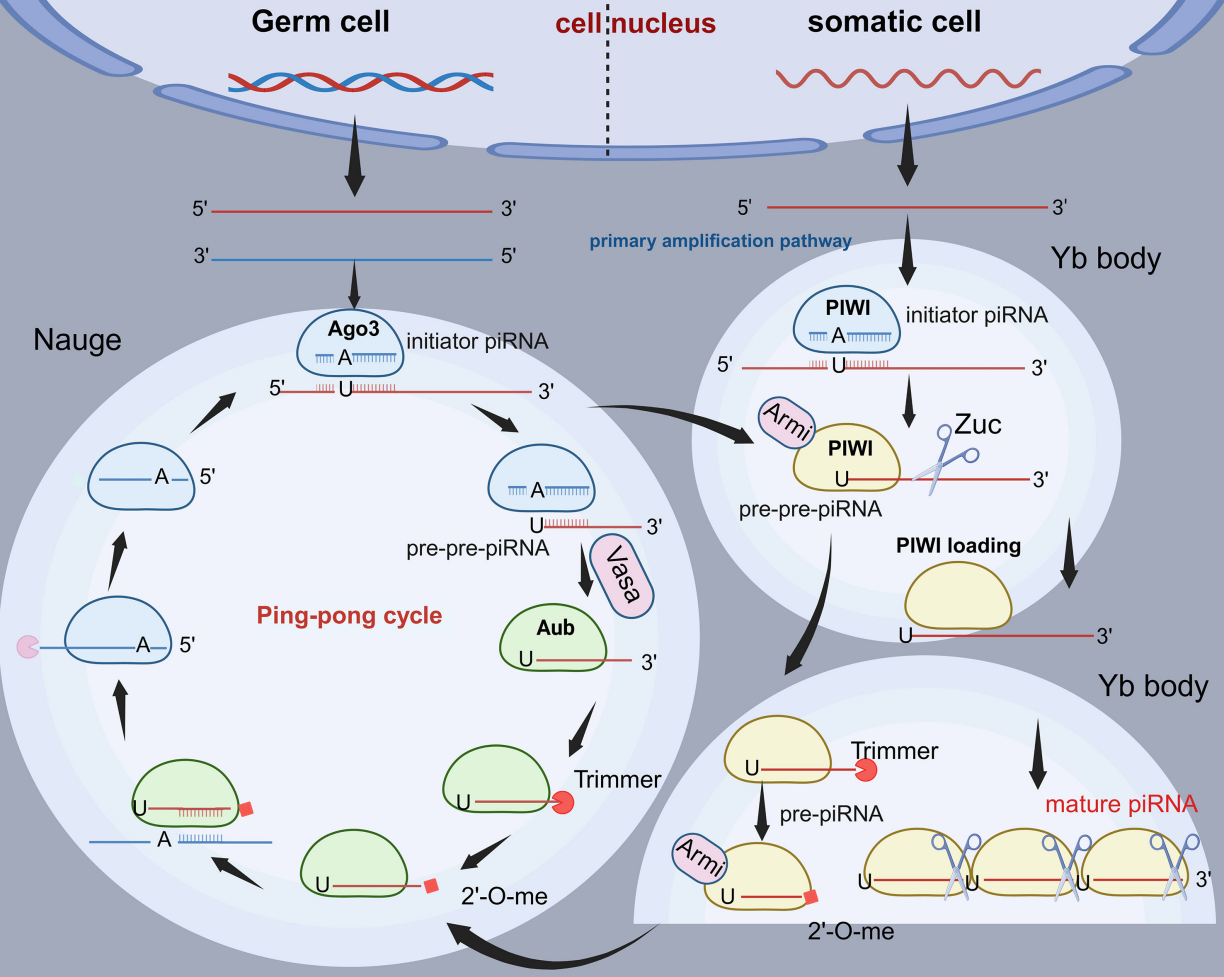
Two modes of piRNA production. In germ cells, Ago3 binds to the initiator piRNA and cleaves the piRNA precursor to generate pre-pre-piRNA, which is guided to Aub by Vasa, and then trimmed by Trimmer and 2’-O-methylated by Hen1 to form a mature piRNA; the mature piRNA binds to complementary precursors and then cleaves the piRNA circularly to generate new pre-pre-piRNA, forming a “ping-pong” cycle. In somatic cells, PIWI binds to the initiator piRNA and cleaves the piRNA precursor into pre-pre-piRNA, which is transferred to the outer membrane of mitochondria by Armi, cleaved by Zuc, trimmed by Trimmer, and then 2’-O-methylated by Hen1 to generate mature piRNA. In addition, primary amplified pre-pre-piRNAs are processed and modified to mature piRNAs upon binding to Armi, thus participating in the “ping-pong” cycling process. Ago3, Argonaute 3; Aub, Aubergine; Vasa, RNA helicase Vasa; Trimmer, exonuclease Trimmer; Hen1, Methyltransferase Hen 1; PIWI, PIWI protein; Armi, Armitage; Zuc, endonuclease Zucchini.

The piRNA precursors are modified to mature piRNAs in the cytoplasm by ribonucleoprotein particles (42, 43). The study shows that piRNA production is highly conserved among species and proposes a unified model (44). The model consists of two linked mechanisms: a primary amplification pathway and a ‘ping-pong’ amplification pathway, and it has been fully validated in the Drosophila (**Figure 2**). In primary amplification, due to the absence of PIWI-like protein Aubergine (Aub) and PIWI-like protein Argonaute 3 (Ago3), the precursor transcription product binds to the Yb body and is cleaved by the internal initiator piRNA to generate early intermediate piRNA (pre-pre-piRNA) (45, 46). Subsequently, it is transferred to the outer mitochondrial membrane by the RNA deconjugating enzyme Armitage (Armi) and cleaved by the endonuclease Zucchini (Zuc), the nucleic acid exonuclease (Trimmer), as well as 2’-O-methylated by the Methyltransferase Hen 1(Hen 1) to obtain mature piRNA (47, 48).

At the beginning of the ‘ping-pong’ cycle, the Ago3-initiator piRNA complex generates a new pre-pre-piRNA by cleaving the transcriptional precursor (49, 50). Subsequently, the molecule is transferred to Aub under the guidance of the RNA helicase Vasa to generate intermediate piRNA (pre-piRNA), and then pre-piRNA is modified by Trimmer and Hen 1 to generate mature piRNA (51, 52). Furthermore, the primary amplified pre-pre-piRNA also participates in the ‘ping-pong’ cycle. It binds to Armi and translocates to the outer mitochondrial membrane, where it is processed and modified to form a mature piRNA (53, 54). Next, the mature piRNA returns to the “ping-pong” cycle as an initiator piRNA that binds and cleaves to the complementary primary transcription precursor to form a new pre-pre-piRNA (16, 55). After binding to Ago3, it undergoes a series of modifications to generate a new piRNA, which has the same sequence as the starting piRNA, thus completing the ‘ping-pong’ cycle (15).

Although this mechanism was first elucidated in Drosophila, accumulating evidence indicates that the fundamental principles of piRNA biogenesis are conserved in mammals, including humans. In mammals, the PIWIL1–4 proteins function similarly to Drosophila’s PIWI, Aub, and Ago3. During spermatogenesis, the primary processing pathway remains dominant, while the ping-pong cycle also contributes to piRNA amplification, despite species-specific modifications (56). These similarities suggest that the Drosophila model provides a valuable framework for understanding piRNA biogenesis in humans and other mammals.

### 2.3 Biosynthesis and cancer

Increasing evidence suggests that the highly conserved piRNA biogenesis pathway may be dysregulated in cancer. For instance, abnormal expression of PIWI proteins (such as PIWIL1 and PIWIL4) in various cancers leads to altered piRNA production and function. Furthermore, auxiliary factors like HENMT1, Arx, and CCR4 play crucial roles in piRNA maturation. Their abnormal expression in tumors leads to defective piRNA biogenesis, impairing both primary processing and the efficiency of the ping-pong cycle. Consequently, this process contributes to the dysregulation of oncogenes and genomic instability. Abnormal piRNA biogenesis not only elucidates the link between piRNAs and cancer initiation and progression but also provides a basis for understanding their multifaceted roles in cancer (57). This topic will be thoroughly examined in the subsequent section.

## 3 Functions of piRNA

Extensive studies have shown that piRNAs maintain the genomic integrity of germ cells by inhibiting transposable element (TE) activity (58). Interestingly, TE silencing is also present in cancer, potentially stabilizing cancer genomes (59). Recent research indicates that piRNAs bind to PIWI to form the piRNA/PIWI complex and regulate target genes through transcriptional silencing and post-transcriptional silencing mechanisms, thereby regulating cancer progression at multiple levels (22). In addition, the complex can be regulated through interactions with a variety of proteins (60). These regulatory network affects cancer development and progression at multiple levels.

### 3.1 piRNA/PIWI complexes mediate transcriptional gene silencing

**Figure 3:**
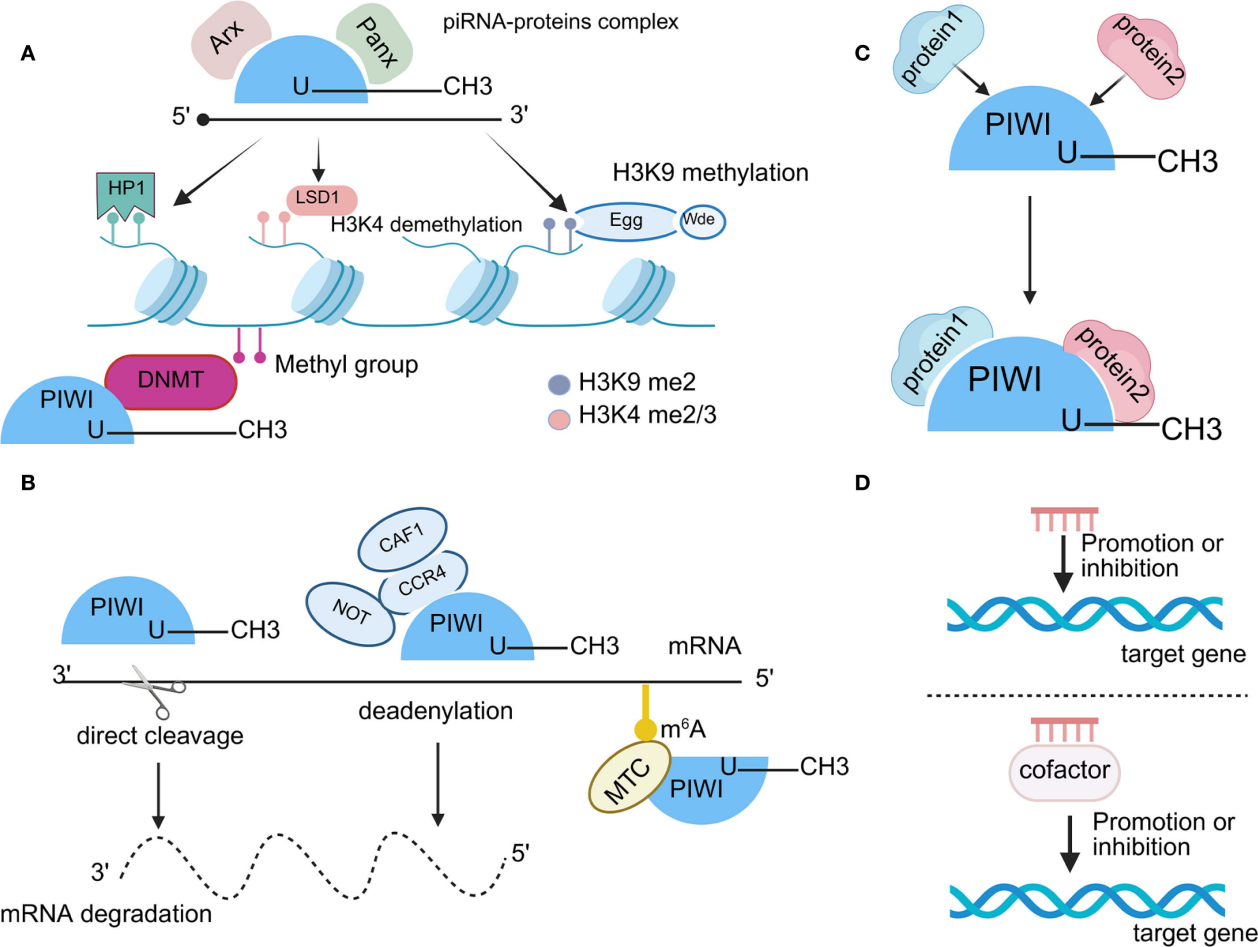
Functions of piRNAs. **(A)** The piRNA/PIWI complex induces TGS by binding to Arx and Panx and targeting new transcripts. Egg and Wde mediate H3K9me2/3 labeling, HP1 recruitment promotes heterochromatin formation, Lsd1 removes H3K4me2 activation markers, and DNMT induces CpG methylation, which collectively represses transcription. **(B)** The piRNA/PIWI complex mediates PTGS by binding to target mRNAs or promoting their deadenylation, leading to mRNA degradation. In addition, m^6^A methylation also participates in PTGS. **(C)** The piRNA/PIWI complex interacts directly with polyproteins. **(D)** piRNAs regulate target genes by binding to cofactors or direct binding genes.

Transcriptional gene silencing (TGS) mediated by the piRNA/PIWI complex occurs primarily at the chromatin level, involving DNA methylation and histone modifications that promote heterochromatin formation and transcriptional repression (**Figure 3A**). This process is initiated when the piRNA/PIWI complex interacts with Asterix (Arx) and Panorama (Panx), recognizing nascent transcripts and anchoring them to specific genomic loci, thereby triggering TGS (61). A key component of this silencing mechanism is histone H3 lysine 9 di- and tri-methylation (H3K9me2/3), catalyzed by the histone methyltransferase Eggless (Egg) and its cofactor Windei (Wde) (62). The deposition of these repressive marks facilitates the recruitment of heterochromatin protein 1 (HP1), which further reinforces heterochromatin architecture and stabilizes transcriptional repression (63). Additionally, the removal of the activating H3K4me2 mark by lysine-specific demethylase 1 (Lsd1) promotes DNA methylation, thereby preventing RNA polymerase II (RNA Pol II) from initiating transcription (64). Beyond histone modifications, the piRNA/PIWI complex also recruits DNA methyltransferases (DNMTs) to CpG sites, leading to DNA methylation and further consolidating transcriptional silencing (64, 65). Research indicates that PIWIL1 methylates the Gem Interacting Protein (GMIP) gene by the RASSF1C-PIWIL1-piRNA signaling axis, leading to down-regulation of the expression of this gene, thereby enhancing NSCLC progression (62).

### 3.2 piRNA/PIWI complexes mediate post-transcriptional gene silencing

In contrast, post-transcriptional gene silencing (PTGS) regulates gene expression at the RNA level, affecting mRNA stability and translation efficiency (**Figure 3B**). Research shows the piRNA/PIWI complex can regulate post-transcriptional gene networks through piRNA-RNA interactions (66). These RNAs include mRNAs (67), long-chain non-coding RNAs (68), and small interfering RNAs. piRNA binds to PIWI proteins to form the piRNA-induced silencing complex (piRISC), which further associates with other inhibitory factors to mediate mRNA degradation, ultimately executing PTGS (69). To illustrate, the piRNA/PIWI complex binds to the CCR 4-NOT complex to form a specific piRISC, which promotes RNA silencing (70). In mouse muscle, piRISC recruits CAF 1, resulting in extensive mRNA degradation and thus facilitating spermatogenesis (71). A study demonstrated that piR-55490 forms incomplete base pairing with the mTOR 3′-UTR, which results in the cleavage and degradation of mTOR mRNA and subsequent inhibition of NSCLC progression (17). Furthermore, piR-211106 was also found to inhibit pyruvate Carboxylase (PC) expression at the mRNA level, which inhibits NSCLC progression. Recent studies have demonstrated that piRNAs can participate in post-transcriptional gene regulation through m^6^A RNA methylation, which is one of the epigenetic regulatory mechanisms currently receiving much attention (72). Notably, this modification requires the specific binding of the corresponding methylase or demethylase post such as m^6^A methyltransferase complex (MTC), to exert a regulatory function on mRNA. For example, piR-27222 inhibited Casp8 expression by enhancing the level of m^6^A methylation modification of Casp8 mRNA through up-regulation of Wilms’ Tumor 1-Associated Protein (WTAP) expression. This process enhanced PANoptosis resistance, which in turn promoted the proliferation and invasion of NSCLC cells (73).

### 3.3 piRNA/PIWI complexes interact with proteins

The third major regulatory mechanism of the piRNA/PIWI complex involves direct interaction with multiple proteins and regulation of downstream pathways (**Figure 3C**). To illustrate, the piR-54265/PIWIL2 complex interacts with signal transducer and activator of transcription 3 (STAT3) and phosphorylated-SRC (p-SRC), mediating SRC phosphorylation via the PAZ structural domain of PIWIL2, which in turn leads to STAT3 phosphorylation activation. This process not only promotes colorectal cancer (CRC) cell tumourigenesis but is also associated with enhanced chemotherapy resistance. Similarly, piR-L-138 can effectively inhibit cisplatin-induced apoptosis by binding to p60 isoform of Mouse double minute 2 homolog (p60-MDM2), a mechanism that significantly contributes to the development of chemoresistance in NSCLC (74). In addition, study reveals that piR-211106 can directly interact with Pyruvate Carboxylase (PC) protein and affect its function at the protein level, thus inhibiting the progression of NSCLC (75).

### 3.4 Other function

The majority of piRNAs exert their gene regulation function in the form of piRNA/PIWI complexes. However, it has been shown that piRNA can also bind to other co-factors to form complexes that can lead to the silencing of target genes or the activation of transcription (**Figure 3D**). In addition, piRNA can directly bind to target genes and inhibit them in a sequence-specific manner (76). For example, piR-L-163 is closely associated with NSCLC, binds directly to phosphorylated ERM protein (p-ERM), and affects p-ERM binding to filamentous actin (F-actin) and ERM-binding phosphoprotein 50 (EBP 50), which consequently affects cell migration and invasive capacity (77). Recent studies have shown that piRNA-PMLCPIR binds to nucleolin (NCL) and promotes the upregulation of integrin subunit beta 1 (ITGB1) expression, which in turn activates the PI3K/AKT signaling pathway and inhibits apoptosis, thereby decreasing the sensitivity of NSCLC to chemotherapy (78).

## 4 piRNAs and PIWI in NSCLC

**Figure 4:**
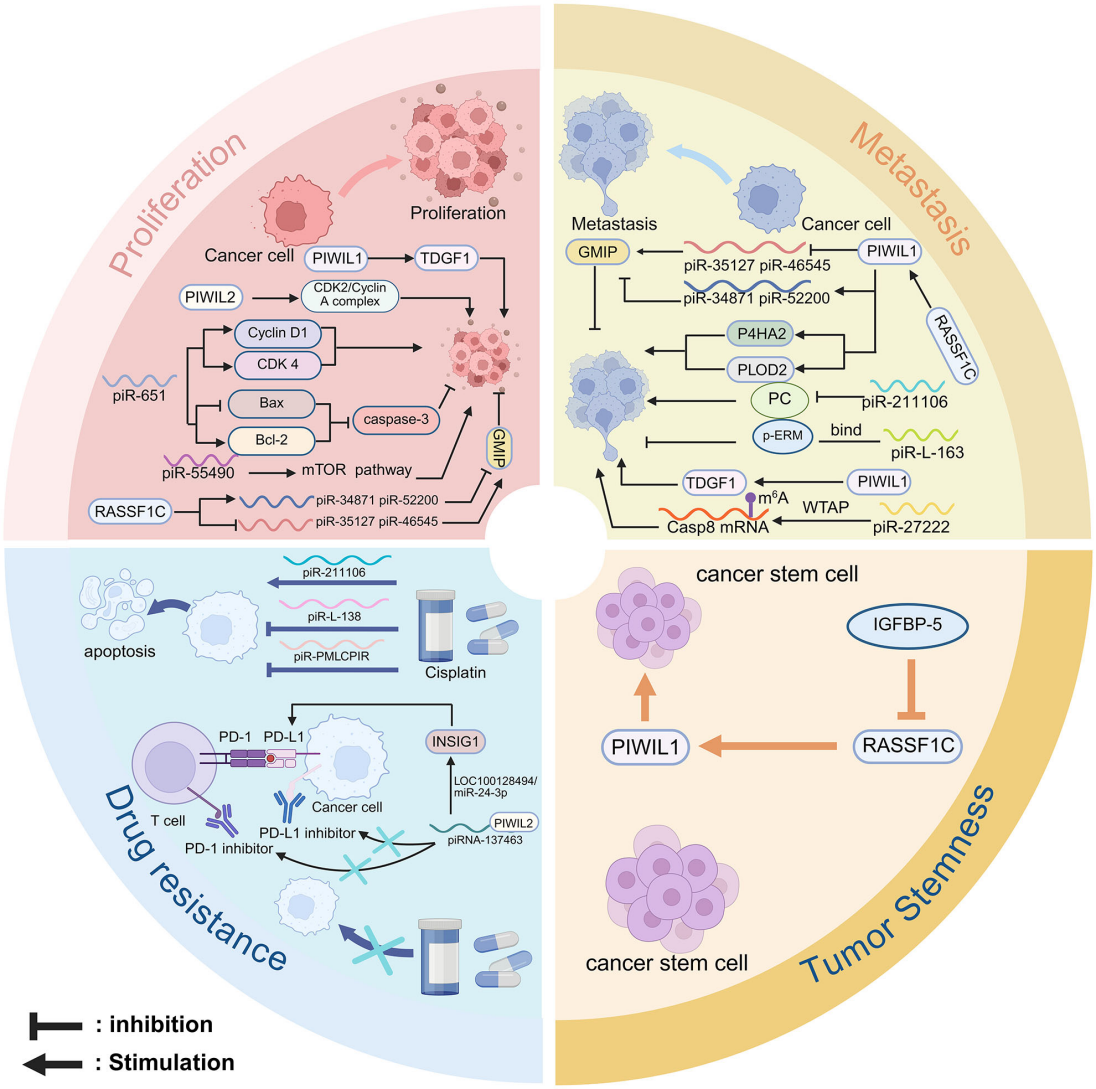
The role of the piRNA and PIWI in NSCLC. Proliferation: PIWIL1 promotes proliferation by activating TDGF1 and the CDK2/Cyclin A complex; PIWIL2 enhances CDK2/Cyclin A complex. piR-651 upregulates Cyclin D1, CDK4, and Bcl-2, and downregulates Bax and caspase-3. piR-55490 activates the AKT/mTOR pathway via mTOR targeting. RASSF1C increases PIWIL1, piR-34871, and piR-52200, and suppresses piR-35127 and piR-46545 to promote proliferation. Metastasis: PIWIL1 enhances migration and invasion through TDGF1. RASSF1C reduces GMIP and increases P4HA2 and PLOD2 expression by PIWIL1. piR-35127 and piR-46545 inhibit metastasis, while piR-211106 suppresses migration by targeting PC. In contrast, piR-L-163 promotes metastasis via p-ERM regulation. piR-27222 upregulates WTAP, increases m6A modification of Casp8 mRNA, thereby inhibiting PANoptosis and enhancing proliferation and invasion. Drug resistance: piR-211106 enhances cisplatin-induced apoptosis and sensitivity. piR-L-138 inhibits apoptosis and promotes cisplatin resistance via the p60-MDM2 axis. piRNA-PMLCPIR upregulates ITGB1, activates the PI3K/AKT pathway, and reduces cisplatin sensitivity. The piRNA-137463/PIWIL2 complex downregulates INSIG1 via the LOC100128494/miR-24-3p axis, promoting cholesterol synthesis and PD-1/PD-L1 upregulation, which enhances immune evasion and resistance to Immune checkpoint inhibitors. Tumor stemness: PIWIL1 enhances stemness via Wnt signaling; IGFBP-5 co-expressed with RASSF1C attenuates this effect by inhibiting PIWIL1.

Research shows that the deregulation of piRNA and PIWI proteins plays a vital role in the pathological mechanisms of NSCLC (79). Despite a variety of available therapeutic approaches, tumor progression remains unaddressed in the clinic. Notably, tumor progression consists of four major biological features proliferation, metastasis, drug resistance, and tumor stemness (60). Numerous studies have confirmed the close association between a variety of piRNA/PIWI proteins and cancer progression (80). Further studies have investigated the oncogenic and suppressive roles of these molecules in the four major features (**Table 1**) (61). Therefore, the part will describe the mechanisms by which piRNAs and PIWI proteins regulate the proliferation, metastasis, chemoresistance, and tumour stemness of NSCLC (**Figure 4**).

**Table 1:** piRNA/PIWI proteins in NSCLC.

piRNA/PIWI	Expression	Related genes/proteins	Region of action	Functions	Regulatory mechanism	Ref
PIWIL1	UP	TDGF1	Cytoplasm	proliferation, Migration and invasion	activating TDGF1	(81)
	UP	RASSF1C, GMIP	Nucleus	proliferation, Migration and invasion	down-regulating GMIP by DNA methylation	(82, 83)
	UP	P4HA2, PLOD2	Nucleus	Migration and invasion	Up-regulating P4HA2 and PLOD2	(84)
	UP	IGFBP-5, RASSF1C, β-catenin	Cytoplasm	Stemness	regulating Wnt signaling pathway to inhibit β-catenin degradation	(76, 85)
PIWIL2	UP	CylinA, CDK2	Cytoplasm	proliferation, apoptosis	Up-regulating the CDK2/Cyclin A complex	(86)
	UP	LOC100128494, miR-24-3p, INSIG1	Cytoplasm	Drug-resistance	promoting upregulation of PD-1/PD-L1 by the LOC100128494/miR-24-3p/INSIG1 pathway	(87)
piR-651	UP	CylinD1, CDK4, Bax, Bcl-2, Caspace-3	Cytoplasm	proliferation, apoptosis	up-regulating Cyclin D1, CDK4, and Bcl-2 and down-regulating Bax and caspase-3	(19, 88)
piR-55490	DOWN	mTOR	Cytoplasm	proliferation	activating the AKT/mTOR pathway	(89)
piR-35127	DOWN	RASSF1C, GMIP	Nucleus	proliferation, Migration and invasion	down-regulating GMIP by DNA methylation	(22, 62, 84)
piR-46545	DOWN	RASSF1C, GMIP	Nucleus	proliferation, Migration and invasion	down-regulating GMIP by DNA methylation	(22, 62, 84)
piR-34871	UP	RASSF1C, GMIP	Nucleus	proliferation, Migration and invasion	down-regulating GMIP by DNA methylation	(22, 62, 84)
piR-52200	UP	RASSF1C, GMIP	Nucleus	proliferation, Migration and invasion	down-regulating GMIP by DNA methylation	(22, 62, 84)
piR-27222	UP	WTAP, PANoptosi, Casp8 mRNA	Cytoplasm	proliferation, Migration and invasion	Inhibiting PANoptosis by increasing Casp8 mRNA methylation	(73)
piR-2111106	DOWN	PC	Cytoplasm	Migration and invasion, Drug-resistance	targeting PC mRNA or proteins	(75)
piR-L-163	DOWN	p-ERM, F-actin, EBP 50	Cytoplasm	Migration and invasion	regulating p-ERM and affecting it binding to F-actin and EBP 50	(77)
piR-L-138	UP	p60-MDM2	Cytoplasm	Drug-resistance	binding to the p60-MDM2 axis	(74)
piR-57125	DOWN	–	–	Migration and invasion	–	(90)
piR-137463	UP	LOC100128494, miR-24-3p, INSIG1	Cytoplasm	Drug-resistance	promoting upregulation of PD-1/PD-L1 by the LOC100128494/miR-24-3p/INSIG1 pathway	(87)
piR-PMLCPIR	UP	NCL, ITGB1, PI3K/AKT	Cytoplasm	Drug-resistance	activating the PI3K/AKT pathway by up-regulating ITGB1	(78)

### 4.1 piRNAs and PIWI in cancer proliferation

Cancer cells exhibit anti-aging and infinitely proliferative properties. Tumor proliferation is achieved through the regulation of the cell cycle, induction of angiogenesis, inhibition of apoptosis and modulation of cell metabolism. Evidence indicates that the abnormal expression of PIWI proteins and piRNAs can disrupt these regulatory mechanisms, thereby promoting the sustained proliferation of NSCLC.

Xie et al. (81) have observed that the activation of PIWIL1 enhances the sensitivity to teratoma-derived growth factor 1 (TDGF1), which combines and participates in downstream signaling pathways, thereby promoting the proliferation of NSCLC cells. It is noteworthy that the high expression of PIWIL1 is not attributable to gene mutation, but rather results from a reduction in the level of DNA methylation in its promoter region. Furthermore, RAS-associated structural domain family 1 member A splice variant (RASSF1C) has been demonstrated to facilitate lung cancer cell proliferation. It activates the MEK-ERK1/2 signaling pathway, promotes ERK1/2 phosphorylation, and upregulates PIWIL1 gene expression (82). Cell cycle proteins and their coordinated family of dependent kinases (CDKs) represent a key regulatory mechanism of cell cycle progression (91). Qu et al. (86) revealed that PIWIL2 plays a key role in NSCLC cell proliferation. This study showed that overexpression of PIWIL2 in H449 and A549 cell lines significantly increased the expression of the CDK2/Cyclin A complex. This contributed to the transition of the cells from the G1 phase to the S phase, accelerated DNA replication and cell cycle progression, and ultimately induced cell proliferation.

Li et al. (19) demonstrated that piR-651 influences the transition from the G1 to S phase by up-regulating the levels of cell cycle proteins D1 and CDK4. This resulted in a decrease in G0/G1-phase cells and an increase in G2/S-phase cells, thereby inhibiting the proliferation process of NSCLC. Furthermore, piR-651 has been demonstrated to regulate proliferation by affecting mitochondria-related apoptotic signaling pathways. Research demonstrated that piR-651 activates Caspase-3 after transfection to promote the proliferation of A549 and HCC827 cells. The process was achieved by mitochondrial inhibition of reactive oxygen species (ROS), up-regulation of Bcl-2 family proteins, and down-regulation of Bax protein (88). In another study, piR-55490 expression was significantly reduced in NSCLC tissues and cells. This small RNA exerts a silencing effect by binding to the 3′ untranslated region (3′UTR) of its target mTOR, which activates the AKT/mTOR signaling pathway, accelerates cell cycle progression, and enhances the efficiency of glucose absorption, collectively leading to the proliferation of NSCLC cells (89). Furthermore, the overexpression of RASSF1C in NSCLC cells has been observed to affect the expression of specific piRNAs, which in turn regulate downstream signaling pathways and involve in the tumor proliferation process. For instance, in the RASSF1C-overexpressed H1299 cell line, piR-34871 and piR-52200 were observed to be overexpressed, whereas the expression of piR-35127 and piR-46545 was downregulated. By knocking down piR-34871 and piR-52200 as well as overexpressing piR-35127 and piR-46545, tumor cells were found to have a significant decrease in their proliferative capacity (84). Further studies showed that RASSF1C could regulate piRNA expression by blocking AMPK phosphorylation and enhancing epidermal growth factor receptor (EGFR) signaling, thereby affecting the proliferation process of NSCLC cells.

### 4.2 piRNAs and PIWI in cancer metastasis

The local invasion and distant metastasis of tumors represent crucial elements of tumor progression and are closely related to patient prognosis. Evidence suggests that the occurrence of metastasis not only increases the malignancy of the tumor but also has a significant negative impact on the five-year survival rate of patients (92). Studies have shown that piRNA and PIWI proteins are important in the metastasis of NSCLC. Therefore, a deeper understanding of the metastatic mechanisms of PIWI and piRNA is expected to provide the latest research basis for early diagnosis and precision treatment of tumors.

Xie et al. observed that in PIWIL1-overexpressing A549 cells, the transwell experiments demonstrated significant enhancement in cell migration and invasion, indicating that PIWIL1 facilitates the invasion and metastasis of NSCLC cells (81). Furthermore, PIWIL1 interacting with TDGF1 may further enhance tumor metastasis, and both drive the progression of tumor invasion and metastasis through synergistic effects. Recent studies have demonstrated that PIWIL1 regulates the methylation status of the oncogene GMIP through the RASSF1C/PIWIL1/piRNA signaling axis, which in turn affects tumor progression (55, 83). This study indicated that the overexpression of RASSF1C resulted in elevated methylation levels of the GMIP gene, which subsequently inhibited GMIP expression and promoted the metastasis of NSCLC cells to distant regions. Furthermore, RASSF1C has been demonstrated to upregulate the expression of prolyl 4-hydroxylase α-2 (P4HA2) and procollagen-lysine, 2-oxoglutarate 5-dioxygenase 2 (PLOD2) through the PIWIL1-piRNA pathway. This promotes extracellular matrix (ECM) remodeling in NSCLC and enhances tumor invasion and metastasis (84).

Research has demonstrated that piR-651 was highly expressed in NSCLC tissues and its expression level was positively correlated with distant tumor metastasis. The results showed that knockdown of piR-651 inhibited the invasion and metastasis of HCC827 and A549 cells, and down-regulation of piR-651 in 95-D cells also reduced their invasive ability (88, 93). Thus, piR-651 can act as an oncogenic factor and also plays an important regulatory role in tumor metastasis. Daugaard et al. identified significant downregulation of piR-57125 in metastatic lung adenocarcinoma, indicating its potential function as a metastasis suppressor in regulating tumor progression (90). Furthermore, Amaar et al. demonstrated that the expression of piR-35127 and piR-46545 was negatively regulated by RASSF1C/PIWIL1 pathway and associated with enhanced invasion and migration of NSCLC cells (22, 84). This indicates that piR-35127 and piR-46545 may function as tumor-suppressive factors that inhibit metastasis. Pyruvate carboxylase (PC), a vital enzyme that connects glycolysis to the tricarboxylic acid cycle, provides the energy for cell migration. Recent studies have demonstrated that piR-211106, acting as an upstream regulatory molecule of PC, reduces the expression level of its genes or proteins by targeting PC mRNA or directly interacting with PC proteins, thereby suppressing the malignant migration phenotype of lung adenocarcinoma cells (75). Another study showed that piR-27222 inhibited Casp8 expression by directly binding to Eukaryotic translation initiation factor 4B (eIF4B) and promoting WTAP expression, which then enhanced the m6A methylation level of Casp8 mRNA. This process inhibited cell death caused by PANoptosis, thereby promoting the proliferation and invasion of NSCLC (73). Notably, the piRNA-like small RNAs (piRNA-Ls), a class of short-stranded non-coding RNAs with structural and functional similarities to piRNAs. They has expanded the diversity of the piRNA family and offers a novel perspective for the understanding of the functional mechanisms of piRNAs (77, 94). There is evidence to suggest that piRNA-Ls are expressed in NSCLC cells and play a role in promoting tumor progression. For instance, Mei et al. demonstrated that piR-L-163 is closely associated with NSCLC and can directly bind to the phosphorylated ERM protein (p-ERM), thereby regulating the functional activity of ERM, which in turn affects the migratory and invasive capacity of cells (77).

### 4.3 piRNAs and PIWI in drug resistance

Drug resistance is closely linked to poor clinical prognosis, which often leads to treatment failure and ultimately to patient death. Therefore, reducing the patient’s drug resistance response is important for improving the efficacy and prolonging the patient’s survival time. Recent studies have shown that piRNAs play a vital role in lung cancer progression and are critical in cancer drug resistance. Specifically, piRNA promotes drug resistance by inhibiting chemotherapy-induced apoptosis or inhibiting immune targeting by PD-1/PD-L1 inhibitors. In addition, PIWI proteins play an important regulatory role in NSCLC drug resistance. In recent years, intensive studies on the functions and mechanisms of piRNA/PIWI proteins have provided new insights into the understanding of drug resistance. Next, we will focus on the specific roles and mechanisms of piRNA/PIWI in NSCLC drug resistance.

It has been demonstrated that piR-211106 remarkably enhances the sensitivity of tumor cells to cisplatin (CDDP), thereby inhibiting tumor progression (75). This phenomenon may be related to the ability of piRNA to enhance chemotherapy-induced apoptosis. Furthermore, piR-211106 exerts a synergistic effect with cisplatin, thereby enhancing the efficacy of the drug. Its use as an adjuvant treatment can effectively inhibit tumor growth when chemotherapy is ineffective. In other chemotherapy resistance study of NSCLC, it was found that piR-L-138 is upregulated in cisplatin-treated NSCLC cells and xenograft mouse models (74). Subsequently, this study observed a significant increase in apoptosis by knocking down piR-L-138 expression. Upon further validation, the upregulated piR-L-138 in p53 mutant Lung squamous cell carcinoma(LSCC) was found to bind directly to the p60-MDM2 axis, inhibiting the cisplatin-induced apoptotic pathway and enhancing chemoresistance (74). This indicates that piR-L-138 may contribute to chemoresistance by inhibiting the apoptotic pathway. Furthermore, Xu et al. found that piRNA-PMLCPIR upregulated ITGB1 expression by binding to NCL and inhibiting the interaction between NCL and ITGB1. This process resulted in activating the PI3K/AKT signalling pathway and inhibiting apoptosis, thereby reducing chemosensitivity to cisplatin (78). In addition to influencing chemotherapy resistance, recent studies have shown that piRNA/PIWI proteins can contribute to the emergence of resistance to PD-1/PD-L1 inhibitors by modulating lipid metabolism. Specifically, the piRNA-137463/PIWIL2 complex reduces INSIG1 expression by regulating the LOC100128494/miR-24-3p/INSIG1 pathway, which then promotes cholesterol synthesis and the upregulation of PD-1/PD-L1 (87). Elevated levels of PD-1/PD-L1 enhance the immune evasion of tumor cells and diminish the therapeutic effect of immune checkpoint inhibitors, thus leading to the development of immune resistance.

### 4.4 piRNAs and PIWI in cancer stemness

Cancer stem cells (CSCs) are critical in oncogenesis, progression and recurrence, and the regulatory mechanisms of their stemness characteristics have become a hotspot in cancer research. Recent studies have confirmed the involvement of the piRNA/PIWI protein complex in the maintenance and regulation of tumour stemness, thereby providing a theoretical basis for targeted therapeutic strategies against CSCs.

PIWIL1, a gene associated with tumor stem cells, is significantly expressed in NSCLC and regulates tumor stem cell proliferation by activating the Wnt signaling pathway to inhibit β-catenin degradation. In addition, the overexpression of RASSF1C upregulates PIWIL1 (85). However, when RASSF1C is co-expressed with insulin-like growth factor-binding protein-5 (IGFBP-5). It regulates PIWIL1 through a post-transcriptional mechanism and potentiates the inhibitory effect on PIWIL1, thus attenuating tumour stemness (76). Currently, the study of piRNAs in tumor stemness in NSCLC is still in the preliminary exploration stage. Therefore, further exploration of the regulatory mechanism of piRNA on cancer stemness will become the highlight of future research.

Taken together, piRNA and PIWI proteins exhibit duality in the malignant phenotype of NSCLC. They not only act as oncogenic factors promoting tumour progression but also exert tumour-suppressing functions under specific conditions. Oncogenic piRNAs, such as piR-651, piR-L-138, and PIWIL2, enhance malignant traits including proliferation, metastasis, drug resistance, and stemness. In contrast, tumor-suppressive piRNAs, such as piR-35127, piR-211106, and piR-46545, restrain these processes and may counterbalance oncogenic signals.The dual role of piRNA/PIWI appears to be subject to multiple regulatory mechanisms, including cofactor interactions, signaling network modulations, and epigenetic modifications. Consequently, this mechanism reveals the intrinsic complexity of piRNAs in NSCLC and broadens its potential as a therapeutic target.

## 5 Potential of piRNAs and PIWI as NSCLC biomarkers

Given the limitations of current biomarkers in terms of sensitivity and specificity,it has led to the limited impact on therapeutic efficacy in NSCLC. Therefore, the exploration of novel tumor markers is particularly important for early diagnosis and efficacy assessment. Evidence shows piRNAs are aberrantly expressed in tumor patients and act as key molecules in signal transduction, playing a vital role in a multitude of regulatory networks (76). It suggests that piRNA has the potential to be a diagnostic and prognostic biomarker (95). And evidence suggested that the high expression of piRNA in exosomes further enhances its potential application in liquid biopsy (96–98). Furthermore, PIWI proteins associated with tumor stemness are able to upregulate the expression of stem cell-related genes and may be new potential biomarkers. This section presents a summary of recent research advances in the field of biomarkers for piRNAs and their interacting PIWI proteins.

Xie et al. postulated that PIWIL1 may act as an epigenetic gene in lung adenocarcinoma, which is associated with lower Recurrence-free survival (TTR) and overall survival (OS), indicating that it can be used as a prognostic marker (81). Recent studies have shown that PIWIL1 expression is regulated by DNA methylation, silenced by hypermethylation in normal lung tissues whereas reactivated by demethylation in NSCLC tissues (21). Furthermore, multivariate analysis showed that high expression of PIWIL1 was an independent poor prognostic marker for NSCLC. Li et al. showed that piR-164586 expression was significantly elevated in exosomes from early-stage NSCLC patients and exhibited high sensitivity and specificity (99). This piRNA was not only stable, but also exhibited a notable decline in expression levels following surgery, thereby demonstrating strong predictive properties for the prognosis of surgical patients. Furthermore, exosomal piR-164586, derived from the serum of NSCLC patients, is anticipated to be applied in the early diagnosis and postoperative monitoring of NSCLC as a novel biomarker with excellent diagnostic and prognostic value (99). Recent studies have demonstrated that exosomal piR-26925 and piR-5444 are significantly expressed in NSCLC. And the results of the ROC curves demonstrated that the Area under the curve(AUC) for both was 0.751 and 0.713, respectively, confirming their efficacy in diagnostic applications (100). Notably, the combination of these two piRNAs demonstrated a more pronounced predictive value, with an AUC of 0.833 (sensitivity = 87.1%, specificity = 75.4%). This predicts a promising application of serum exosome piR-26925 and piR-5444 in NSCLC diagnosis (100). In addition, piR-651 was found to play a pro-oncogenic role in NSCLC through a mechanism that may be related to up-regulation of the expression of the cell cycle protein D1/CDK4, suggesting that piR-651 could be used as a potential diagnostic indicator for NSCLC (19). Conversely, piR-55490 inhibited the activation of the Akt/mTOR pathway by binding to the 3’UTR of mTOR mRNA, thereby prolonging the survival time of NSCLC patients and demonstrating its value in predicting the prognosis of NSCLC (89). And with the advancement of second-generation RNA transcriptome sequencing technology, the discovery of novel piRNA markers will be easier. However, the research progress of PIWI protein in the field of NSCLC biomarkers is relatively slow. Therefore, future studies should focus on exploring its potential as a biomarker, revealing its diagnostic and prognostic assessment value through systematic analyses and clinical validation, and providing new ideas for the treatment of NSCLC.

## 6 piRNAs and PIWI as potential targets in NSCLC

The incidence and mortality of NSCLC have remained high in recent years, and this can be attributed to limitations in diagnostic and therapeutic tools (5). Consequently, the development of novel therapeutic strategies is of particular importance. Much research indicates that piRNA/PIWI proteins may offer new therapeutic strategies as potential therapeutic targets for NSCLC.

PIWI proteins have the potential to serve as therapeutic targets due to their unique protective mechanisms and lower toxicity. Studies have shown that in NSCLC tumor cells, the PIWIL1 promoter region promotes its expression by recruiting demethylases. Therefore, epigenetic regulatory drugs such as DNA methylation inhibitors and Histone deacetylase (HDAC) inhibitors can be used to intervene in the expression of PIWIL1 and thus affect the progression of NSCLC. The finding suggests that PIWIL1 may be a new therapeutic target (21). Another study demonstrated that PIWIL2 plays a role in reducing tumor progression induced by DNA damage by participating in the process of DNA repair (86). Therefore, PIWIL2 is expected to be a new targeted drug with fewer side effects. Furthermore, findings demonstrate that piRNAs have a stable delivery capacity and can act as oncogenes or suppressor oncogenes to regulate downstream signaling factors involved in tumorigenesis and progression, thus becoming new therapeutic targets for NSCLC (101). In particular, piRNA-targeted therapy inhibits specific oncogenic processes by regulating the expression of aberrant piRNAs, affecting both the pre- and post-transcriptional levels of genes and proteins, and thus preventing further tumor progression. Although the intricate signal regulatory network and potential for off-target effects of piRNAs present challenges for the development of targeted drugs, the great potential of piRNA-targeted therapeutics is expected to solve this dilemma (76). For example, In a mouse xenograft tumor model, tumor formation and progression were accelerated in mice following transplantation of A549 cells overexpressing piR-651. It suggests that piR-651 has potential as a therapeutic target for NSCLC (19). Wang et al. demonstrated that the down-regulation of piR-L-138 expression increased the sensitivity of lung squamous cell carcinoma cells to cisplatin, suggesting that the combination of piRNA-targeted therapy and chemotherapy may result in a more significant therapeutic effect (74). Currently, immunotherapy has made remarkable progress in tumor treatment. In addition, with the development of high-throughput sequencing and bioinformatics technologies, future studies need to be devoted to the discovery of novel piRNAs and PIWI proteins and to the in-depth investigation of their effectiveness and clinical significance as therapeutic targets for NSCLC.

In recent years, research has focused on piRNA mimics and inhibitors as emerging targeted therapeutic strategies. In tumors, oncogenic piRNAs can accelerate tumor progression by promoting cell proliferation, metastasis, or inducing chemo-resistance; inhibitors targeting these piRNAs have been shown to inhibit them in some tumor models. For example, piRNA-17458 inhibitor suppresses the malignant phenotype of Cervical Cancer (CC) cells (102). Conversely, downregulated tumour-suppressing piRNAs can have their expression restored by piRNA mimics, thereby enhancing anti-tumour efficacy. However, the study of tumour-suppressing piRNA mimics is still relatively limited, especially in NSCLC. Nonetheless, this strategy demonstrates theoretical potential and offers novel insights for targeted therapies in NSCLC.

## 7 Conclusion and prospects

NSCLC is one of the leading causes of cancer-related deaths globally and is usually diagnosed at an advanced stage. Therefore, various treatments, including surgery, chemotherapy and targeted therapies, have limited efficacy. Recent studies have demonstrated the pivotal function of the piRNA/PIWI complex in the pathogenesis and progression of cancer. In physiological conditions, piRNAs and PIWI proteins are maintained in stable equilibrium. However, when this equilibrium is disrupted, it may contribute to tumour formation. The advent of second-generation sequencing technology has facilitated considerable advancement in the identification of functional piRNA/PIWI proteins in tumour tissues. Therefore, piRNAs and PIWI hold potential clinical application value in various cancers. In diagnostics, piRNAs and PIWI proteins can be detected in tissues and circulating biofluids, often exhibiting superior specificity and sensitivity compared to traditional biomarkers. In prognostic assessment, abnormal expression of certain piRNA/PIWI correlates closely with patient overall survival and disease recurrence risk. Furthermore, functional studies indicate that modulating specific piRNAs or PIWI proteins significantly impacts tumor growth, metastasis, and treatment response, suggesting their potential as therapeutic targets. This provides a broader context for understanding the role of piRNA/PIWI in NSCLC. This review first describes the biological origin and function of the piRNA and PIWI proteins. Specifically, piRNA binds to PIWI proteins and regulates the expression of target genes at the transcriptional level, for example by affecting histones and DNA methylation. Furthermore, piRNAs operate through post-transcriptional regulatory mechanisms, such as mRNA shearing and m6A methylation, or through interactions with multiple proteins. This review further explores the effects of piRNA and PIWI proteins on four major tumour phenotypes, including proliferation, metastasis, stem cell properties and chemotherapy resistance. To illustrate, piR-651 has been demonstrated to influence the proliferation, invasion and metastatic capacity of NSCLC through multiple molecular pathways. Notably, PIWI proteins can regulate tumour progression and metastasis independently of interaction with piRNA. Combined with recent studies, we discuss the potential value of piRNA/PIWI complexes as biomarkers and therapeutic targets for NSCLC. A large number of piRNAs and PIWI proteins were found to be abnormally expressed in blood with specificity and sensitivity superior to existing indicators, indicating their potential as biomarkers. In conclusion, there is growing evidence that piRNAs and PIWIs play a key role in epigenetic regulation and cancer phenotypes and have great potential for diagnosing and treating tumours. Therefore, the regulatory mechanisms of the piRNA and PIWI proteins in NSCLC development should be further investigated in the future to gain a more comprehensive understanding. Meanwhile, the specific role of the complex as a tumour regulator in the tumour microenvironment and metabolic regulation needs to be clarified. In addition, the discovery of novel piRNAs and PIWI proteins and deeper investigation of their feasibility and clinical significance as biomarkers and therapeutic targets for NSCLC are important directions for future research.

However, the application of piRNA and PIWI proteins in NSCLC still faces some challenges. Firstly, existing studies on the understanding of the piRNA and PIWI proteins have been mainly based on biological models, and the evidence for human and mammalian models has been lacking. In addition, studies of biological functions have mostly focused on transcriptional and post-transcriptional level regulation, while mechanisms at the translational level have been less studied. With regard to the clinical aspect, research results as a therapeutic target are mostly limited to combination with conventional chemotherapeutic agents, and its modulating role in immunotherapy and radiotherapy is yet to be developed. In addition, piRNA/PIWI-targeted therapies still suffer from off-target effects due to binding to non-target RNA, the lack of an efficient and tissue-specific delivery system, and differences in therapeutic specificity due to individual heterogeneity of patients. These issues may affect the predictability and safety of therapeutic efficacy. In clinical translation, species differences and inconsistencies in experimental conditions may lead to poor reproducibility of preclinical models. In addition, the lack of standardized protocols for piRNA detection and functional validation, as well as the lack of large-scale, multicenter clinical studies, impede successful translation to clinical applications. Moreover, piRNA research continues to face numerous challenges at both the technical and methodological levels. On the one hand, the short length and complex sequences of piRNAs render their detection difficult. On the other hand, functional validation tools remain imperfect, and next-generation high-throughput sequencing technologies have yet to become widely adopted.Despite the multiple challenges, more and more studies are continuously exploring more precise delivery systems to minimize the interference of non-target RNAs. Precision medicine and personalized treatment strategies will also effectively address such issues by addressing individual-specific variations. With the advancement of high-throughput sequencing technology, future multicenter and large sample studies will provide more data support for the clinical application of piRNAs (103). Meanwhile, standardization of data analysis and application of AI will help to rapidly identify the role of piRNA and PIWI and their potential in tumors. In the future, piRNA/PIWI research will hopefully be integrated with multi-omics technologies, especially genomics, transcriptomics, proteomics and metabolomics (104). This contributes to our comprehensive understanding of the functions of piRNAs and their roles in tumors, and provides more effective diagnostic and prognostic value in the clinic. In addition, the rapid development of artificial intelligence and machine learning has provided new opportunities for piRNA biomarker discovery. Through AI analysis of large-scale histological data, researchers can identify potential biomarkers and predict their relationship to tumorigenesis, progression, and response to treatment (105). Meanwhile, advances in exosome-based liquid biopsy technologies provide powerful support for the discovery of PIWI/piRNA-related biomarkers (97, 98). This will not only accelerate the discovery of piRNA and PIWI biomarkers, but also enhance the precision and personalization of existing therapeutic strategies. Therefore,we believe that future studies will further illuminate the biological mechanisms of the piRNA and PIWI proteins and their potential role in the treatment and diagnosis of NSCLC.

## Glossary

NSCLC: Non-small cell lung cancer
SCLC: Small cell lung cancer
piRNA: PIWI-interacting RNA
miRNA: MicroRNA
RISC: RNA-induced silencing complex
TE: Transposable element
TGS: Transcriptional gene silencing
PTGS: Post-transcriptional gene silencing
Pol II: RNA polymerase II
RDC: Rhino-Deadlock-Cutoff
H3K9me3: Histone H3 lysine 9 trimethylation
H3K4me2: Histone H3 lysine 4 dimethylation
CI: Cubitus interruptus
Aub: Aubergine
Ago3: Argonaute 3
Armi: Armitage
Zuc: Zucchini
Hen1: Methyltransferase Hen 1
Vasa: RNA helicase Vasa
Arx: Asterix
Panx: Panorama
H3K9me2: Histone H3 lysine 9 dimethylation
Egg: Eggless
Wde: Windei
HP1: Heterochromatin protein 1
Lsd1: Lysine-specific demethylase 1
DNMT: DNA methyltransferase
GMIP: Gem Interacting Protein
RASSF1C: RAS-associated structural domain family 1 member C splice variant
piRISC: piRNA-induced silencing complex
mTOR: Mammalian target of rapamycin
PC: Pyruvate Carboxylase
m6A: N6-methyladenosine
METTL14: Methyltransferase-like 14
CYP1B1: Cytochrome P450 1B1
CC: Cervical cancer
STAT3: Signal transducer and activator of transcription 3
p-SRC: Phosphorylated-SRC
CRC: Colorectal cancer
MDM2: Mouse double minute 2 homolog
p-ERM: Phosphorylated ERM protein
F-actin: Filamentous actin
EBP 50: ERM-binding phosphoprotein 50
TDGF1: Teratoma-derived growth factor 1
CDK: Cyclin-dependent kinase
MEK: Mitogen-activated protein kinase kinase
ERK1/2: Extracellular signal-regulated kinase 1/2
EGFR: Epidermal growth factor receptor
P4HA2: Prolyl 4-hydroxylase α-2
PLOD2: Procollagen-lysine, 2-oxoglutarate 5-dioxygenase 2
ECM: Extracellular matrix
ROS: Reactive oxygen species
AMPK: Adenosine monophosphate-activated protein kinase
CDDP: Cisplatin
CSC: Cancer stem cell
IGFBP-5: Insulin-like growth factor-binding protein-5
TTR: Recurrence-free survival
OS: Overall survival
AUC: Area under the curve
HDAC: Histone deacetylase
Trimmer: Nucleic acid exonuclease
p60-MDM2: p60 isoform of Mouse double minute 2 homolog
NCL: Nucleolin
ITGB1: Integrin subunit beta 1
eIF4B: Eukaryotic translation initiation factor 4B
LSCC: Lung squamous cell carcinoma
(LLMs): No large language models or other AI tools were used in the generation of this manuscript
